# *ThCOL2* Improves the Salt Stress Tolerance of *Tamarix hispida*

**DOI:** 10.3389/fpls.2021.653791

**Published:** 2021-05-17

**Authors:** Xiaojin Lei, Bing Tan, Zhongyuan Liu, Jing Wu, Jiaxin Lv, Caiqiu Gao

**Affiliations:** State Key Laboratory of Tree Genetics and Breeding, Northeast Forestry University, Harbin, China

**Keywords:** *Tamarix hispida*, COL transcription factor, *ThCOL2* gene, salt stress, ROS-scavenging capability

## Abstract

The CONSTANS-LIKE (COL) transcription factor has been reported to play important roles in regulating plant flowering and the response to abiotic stress. To clone and screen *COL* genes with excellent salt tolerance from the woody halophyte *Tamarix hispida*, 8 *ThCOL* genes were identified in this study. The expression patterns of these genes under different abiotic stresses (high salt, osmotic, and heavy metal) and abscisic acid (ABA) treatment were detected using quantitative real-time PCR (qRT-PCR). The expression levels of 8 *ThCOL* genes changed significantly after exposure to one or more stresses, indicating that these genes were all stress-responsive genes and may be involved in the stress resistance response of *T. hispida*. In particular, the expression level of *ThCOL2* changed significantly at most time points in the roots and leaves of *T. hispida* under salt stress and after ABA treatments, which may play an important role in the response process of salt stress through a mechanism dependent on the ABA pathway. The recombinant vectors pROKII–*ThCOL2* and pFGC5941–*ThCOL2* were constructed for the transient transformation of *T. hispida*, and the transient infection of *T. hispida* with the pROKII empty vector was used as the control to further verify whether the *ThCOL2* gene was involved in the regulation of the salt tolerance response of *T. hispida*. Overexpression of the *ThCOL2* gene in plants under 150 mM NaCl stress increased the ability of transgenic *T. hispida* cells to remove reactive oxygen species (ROS) by regulating the activity of protective enzymes and promoting a decrease in the accumulation of O^2–^ and H_2_O_2_, thereby reducing cell damage or cell death and enhancing salt tolerance. The *ThCOL2* gene may be a candidate gene associated with excellent salt tolerance. Furthermore, the expression levels of some genes related to the ABA pathway were analyzed using qRT-PCR. The results showed that the expressions of *ThNCED1* and *ThNCED4* were significantly higher, and the expressions of *ThNCED3*, *ThZEP*, and *ThAAO*3 were not significantly altered in OE compared with CON under normal conditions. But after 24 h of salt stress, the expressions of all five studied genes all were lower than the normal condition. In the future, the downstream genes directly regulated by the *ThCOL2* transcription factor will be searched and identified to analyze the salt tolerance regulatory network of *ThCOL2*.

## Introduction

The *CONSTANS* (*CO*) gene was first isolated in the Arabidopsis flowering delay mutant, and then 16 *CONSTANS-LIKE* (*COL*) genes were isolated in Arabidopsis, and together, these genes constitute a COL transcription factor family. The COL protein encoded by the *COL* gene contains two homologous domains: the B-box domain (zinc finger domain) located near the N-terminus and the CCT (CO, CO-like, and TOC1) domain located near the C-terminus. The COL family is divided into three groups based on the B-box domain. The first group contains two B-box domains and is further subdivided into five subcategories according to the conserved region between the B-box and CCT domains: Ia, Ib, Ic, Id, and Ie. The second group contains one B-box domain. The third group contains one B-box domain and one zinc finger protein domain with changes in the secondary structure (Putterill et al., 1995; Robson et al., 2001).

In recent years, COL transcription factors have been identified in many plants, such as *Arabidopsis thaliana* (Zhang et al., 2014), *Brassica juncea* (Muntha et al., 2019), *Phyllostachys violascens* (Xiao et al., 2018), and *Oryza sativa* (Sheng et al., 2016). Most COL transcription factors have been reported to be involved in flowering regulation. In Arabidopsis, an increase in the expression of *AtCOL4* leads to a decrease in the expression of *FT* and *APETALA 1* (*AP1*) and subsequently suppresses flowering (Steinbach, 2019). *AtCOL3* inhibits flowering by interacting with *BBX32* to inhibit the transcription of the target gene *FT* (Tripathi et al., 2017). In rice, *OsCOL15* inhibits flowering by promoting *Ghd7* expression and inhibiting *RID1* expression (Wu et al., 2018). *OsCOL10* inhibits flowering by reducing *Ehd1* expression and acts on *Ghd7* (Tan et al., 2016, 2017). *OsCOL9* delays flowering time in rice by inhibiting the *Ehd1* pathway (Liu et al., 2016a).

In addition to the regulation of flowering time, some *COL* genes are also involved in the regulation of plant responses to abiotic stress. Arabidopsis *OMG1* plays a role in regulating the ROS pathway by regulating related genes (*MYB77* and *GRX480*) (Sng et al., 2018). *AtCOL4* participates in salt stress responses through ABA-dependent signaling pathways (Min et al., 2015). Many *ZmCOLs* are activated or inhibited to varying degrees in maize under ABA stress, indicating that *ZmCOLs* may be involved in stress responses related to the ABA pathway (Song et al., 2018). In rice, overexpression of the *Ghd2* gene increases sensitivity to drought (Liu et al., 2016b). In banana, the expression of the *MaCOL1* gene is significantly induced at different times of cold stress and participates in cold stress responses (Chen et al., 2012).

Moreover, the *COL* genes are also involved in other regulatory mechanisms. In apple (*Malus domestica*), the overexpression of *MdBBX1* reduces the ethylene content in transgenic apple strains and changes the anthocyanin concentration and expression of related genes to regulate fruit color (Plunkett et al., 2019). Rice OsCOL9 interacts with OsRACK1 and enhances rice blast resistance through salicylic acid and ethylene signaling pathways (Liu et al., 2016c). Morita et al. (2015) showed that the rice COL protein CRCT is a positive regulator of starch accumulation in vegetative tissues and regulates the coordinated expression of starch synthesis genes in response to photosynthesis. In Arabidopsis, AtCOL7 regulates the branching and shading response (Wang et al., 2013).

*Tamarix hispida* is a woody halophyte with strong salt tolerance. It grows normally in soil with less than 1% salt content. Therefore, it is an ideal material for studying the mechanism of salt tolerance and cloning salt tolerance genes (Gao et al., 2012). In the present study, 8 *COL* genes (named *ThCOL1-8*) were cloned from *T. hispida*, and their expression patterns in response to salt, osmotic, and heavy metal stresses and ABA treatments were analyzed in different *T. hispida* tissues. Furthermore, *ThCOL2* was selected for further study and transiently transformed into *T. hispida*. *ThCOL2* overexpression significantly improved the salt tolerance of transgenic *T. hispida*. This study provides a potential candidate salt tolerance-related gene for tree forestry genetics and breeding.

## Materials and Methods

### Plant Materials and Stress Treatments

The culture conditions of *T. hispida* seedlings were determined according to the methods described by Wang et al. (2019). 2-months-old seedlings were exposed to one of the following solutions for 6, 12, 24, 48 or 72 h: 0.4 M NaCl, 20% (w/v) PEG_6000_, 150 μM CdCl_2_ or 100 μM ABA. At the same time, seedlings irrigated with fresh water were used as controls. After these treatments, the leaves and roots of at least 24 seedlings were harvested for RNA extraction. Three independent treatments were performed.

### Identification of the *ThCOL* Genes in *T. hispida*

Eight *ThCOL* genes were identified from the seven transcriptome libraries (Wang et al., 2014) and confirmed based on a blast search of the conserved domain^1^. Then, the *ThCOL* genes with complete open reading frames (ORFs) were selected using ORF finder^2^. The GenBank accession numbers of ThCOL1-8 were MW846245-846252. The molecular weight and theoretical isoelectric point (pI) of the ThCOL proteins were determined using ProtParam^3^.

### Bioinformatics Analysis of the *ThCOL* Genes

The amino acid sequences of the 8 ThCOL proteins were selected for multiple sequence alignment, and 8 ThCOL proteins and 17 COL proteins in Arabidopsis were selected for the phylogenetic analysis. Multiple sequence alignments were performed using BioEdit software, and an unrooted phylogenetic tree was constructed with MEGA 5.05 software (Tamura et al., 2011).

### RNA Extraction and qRT-PCR

Total RNA was extracted using the Plant total RNA extraction kit (BioTeke Corporation). According to the instructions from the TransScript One-Step gDNA Removal and cDNA Synthesis SuperMix Kit (Trans, Beijing), 1.0 μg of RNA was reverse transcribed to cDNAs, and the cDNAs were diluted 10 times with sterile water used as a template for quantitative real-time PCR (qRT-PCR). β-Actin (FJ618517), α-tubulin (FJ618518) and β-tubulin (FJ618519) were selected as internal controls (Zang et al., 2015). All primers used for qRT-PCR are listed in Supplementary Table 1. The reaction system parameters were the same as those described by Wang et al. (2019), and the relative quantitative analysis of gene expression was performed using the 2^–ΔΔCt^ method (Livak and Schmittgen, 2001).

### Analysis of the Subcellular Location

The full-length ThCOL2 coding region (without the stop codon) was ligated to the 5’ end of green fluorescent protein (GFP) to generate a *ThCOL2*-GFP fusion vector, which was driven by the cauliflower mosaic virus (CaMV) 35S promoter. A 35S:GFP vector was used as the control. All primer sequences used are listed in Supplementary Table 1. The specific operation methods were the same as those described by Liu Z. Y. et al. (2020).

### Analysis of Transactivation Activity

Based on the conserved domain, full-length ThCOL2 was truncated into three fragments. Specifically, the first fragment was the entire N-terminus containing the two B-box domains, the second fragment was the middle region without any conserved domain, and the third fragment was the entire C-terminus containing the CCT domain. The full-length ThCOL2 (1–432 aa) and multiple truncated fragments, dC1 (1–138 aa), dC2 (139–346 aa), dC3 (347–432 aa), dC4 = dC1 + dC2 (1–346 aa), and dC5 = dC2 + dC3 (139–432 aa), were fused to the GAL4 DNA binding domain in the pGBKT7 vector. All primer sequences are listed in Supplementary Table 1. The domain required for transcriptional activation was determined by screening the growth status on SD/-Trp/-His/X-a-Gal media. The specific experimental steps were performed according to the instructions of Matchmaker^TM^ (Gold Yeast Two-Hybrid System).

### Vector Construction and Transient Expression of *ThCOL2* in *T. hispida*

The CDS of *ThCOL2* was inserted into pROKII and driven by the 35S CaMV promoter to overexpress *ThCOL2* (35S:COL). An inverted repeat truncated CDS of *ThCOL2* that was 246 bp in length was inserted into pFGC5941 at the two ends of the CHSA intron (pFGC:COL) to silence *ThCOL2* expression (Supplementary Figure 1). The primers used for this experiment were listed in Supplementary Table 1. The genetic transformation of *T. hispida* plants was performed according to the protocol described by Liu Z. Y. et al. (2020). Three types of transiently transformed plantlets and at least nine seedlings of each type of transgenic *T. hispida* plant were harvested and used for qRT-PCR analysis. Three independent experiments were performed.

### Biochemical Staining and Physiological Measurements of Transformed Plants

After culture for 48 h on 1/2 MS medium, the seedlings from transient transgenic *T. hispida* (OE, IE, and Con) were transferred either to fresh 1/2 MS medium or 1/2 MS supplemented with 150 mM NaCl for 2 h. Then, the seedlings were harvested and incubated with NBT, DAB, or Evans blue solutions. The procedures for NBT and DAB staining were performed using the method reported by Zhang et al. (2011). Evans blue staining was performed as described by Kim et al. (2003). Each sample contained at least nine harvested seedlings, and three independent experiments were performed.

In addition, after culture for 48 h on 1/2 MS medium, the plants were transferred to 1/2 MS medium containing 150 mM NaCl for either 12 or 24 h and then harvested for physiological analyses. POD, electrolyte leakage and MDA measurements were performed using the methods reported by Zang et al. (2015). H_2_O_2_ levels, SOD activity and CAT activity were measured according to the instructions provided with the reagent kit (Nanjing Jiancheng Bioengineering Institute, China). Each sample contained at least 9 seedlings, and three independent experiments were performed. qRT-PCR was used to detect the relative expression levels of five ABA biosynthesis-related genes (*NCED1*, *3*, *4, ZEP*, and *AAO3*) (Tan et al., 2020) in OE transgenic plants after normal condition and stress with 150 mM NaCl for 24 h. The primers used for qRT-PCR are listed in Supplementary Table 1.

### Statistical Analysis

The data were compared using Student’s *t*-test. ^∗^Indicates a significant difference (*P* < 0.05), ^∗∗^represents a very significant difference (*P* < 0.01).

## Results

### Identification of *ThCOL2* Genes in *T. hispida*

In total, eight candidate *COL* genes (named *ThCOL1*–*ThCOL8*) with complete ORFs were identified from the *T. hispida* transcriptome. The total length of the 8 *ThCOL* genes was 1,089–1,467 bp. The number of amino acid residues ranged from 362 to 488, and the predicted molecular weights (MWs) were 39.6–53.6 kDa, with a theoretical isoelectric PI of 4.83–7.48 (Table 1).

**TABLE 1:** Features of *ThCOL* genes in *Tamarix hispida*.

Name	GenBank accession numbers	ORF (bp)	Protein length	Theoretical pI	Molecular weight (kD)
*ThCOL1*	MW846245	1,176	391	4.97	42.12
*ThCOL2*	MW846246	1,296	431	5.78	47.75
*ThCOL3*	MW846247	1,404	467	5.83	51.9
*ThCOL4*	MW846248	1,218	405	5.95	45.5
*ThCOL5*	MW846249	1,089	362	7.48	39.6
*ThCOL6*	MW846250	1,467	488	7.03	53.6
*ThCOL7*	MW846251	1,188	395	4.83	42.9
*ThCOL8*	MW846252	1,137	378	5.49	41.7

*ORF, open reading frame; bp, base pair; aa, amino acid; MW, molecular weight; pI, isoelectric point; kDa, kilodalton.*

### Classification of Eight *ThCOLs*

**FIGURE 1:**
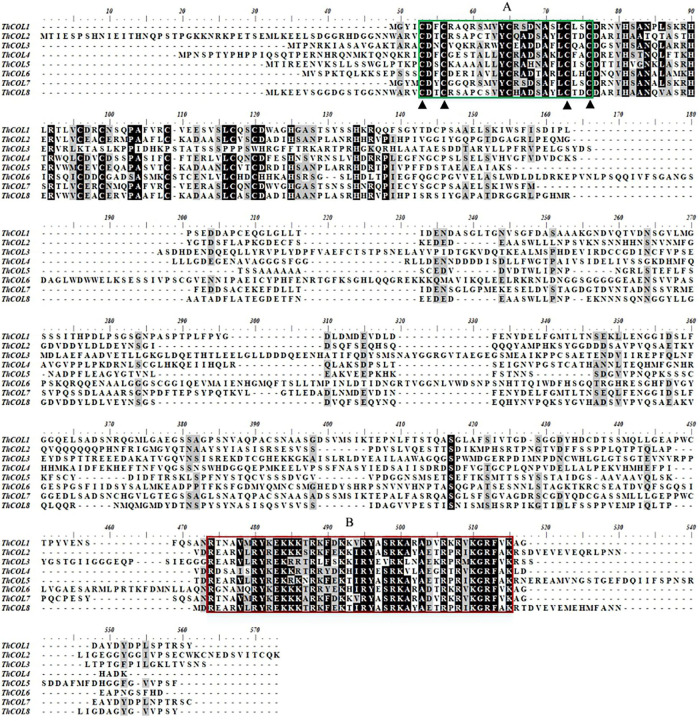
Multiple sequence alignment of 8 ThCOLs protein. **(A)** (Green box) was the first B-box at the N-terminal of COL protein. **(B)** (Red box) was the nuclear localization signal region of COL protein.

**FIGURE 2:**
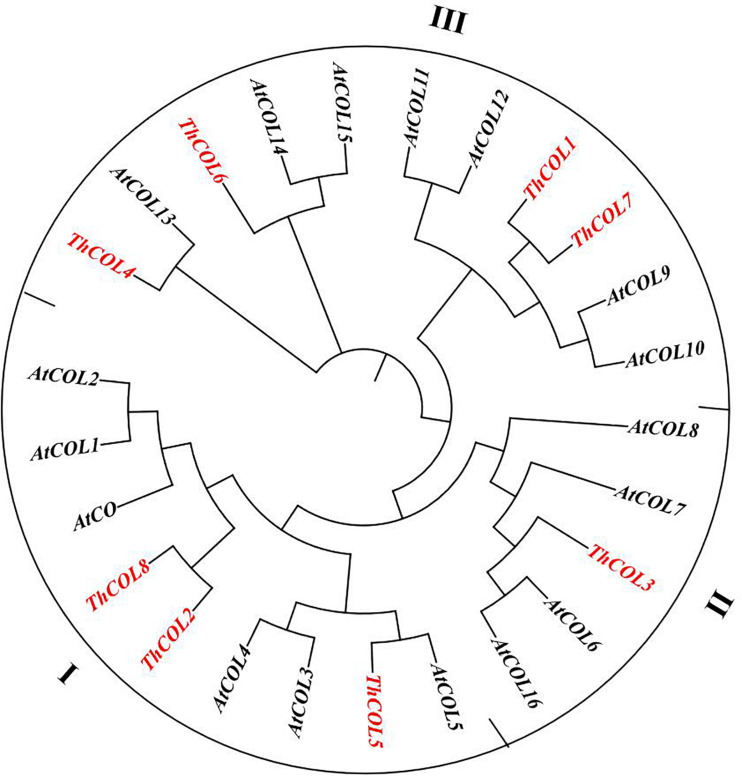
Phylogenetic analysis of ThCOLs. Analysis was performed with both ThCOLs and Arabidopsis proteins. ThCOLs sequences were downloaded from the seven transcriptome libraries. The accession numbers of Arabidopsis sequences in the phylogenetic are: AtCO (Q39057), AtCOL1 (O50055), AtCOL2 (Q96502), AtCOL3 (Q9SK53), AtCOL4 (Q940T9), AtCOL5 (Q9FHH8), AtCOL6 (Q8LG76), AtCOL7 (Q9C9A9), AtCOL8 (Q9M9B3), AtCOL9 (Q9SSE5), AtCOL10 (Q9LUA9), AtCOL11 (O23379), AtCOL12 (Q9LJ44), AtCOL13 (O82256), AtCOL14 (O22800), AtCOL15 (Q9C7E8), and AtCOL16 (Q8RWD0).

Multiple sequence alignments were performed on 8 COL proteins from *T. hispida*. The amino acid identity of the eight ThCOL proteins ranged from 22.83 to 71.95%. The proteins contained a conserved B-box domain at the N-terminus and a CCT domain at the C-terminus (Figure 1). An unrooted phylogenetic tree was constructed using MEGA with full-length protein sequences of *T. hispida* COLs and AtCOLs (Figure 2). The 8 ThCOL proteins clustered into three groups, of which ThCOL2, ThCOL5, and ThCOL8 were closely related, belonging to the first group, and contained two complete B-box domains at the N-terminus. ThCOL3 belonged to the second group, which contained a complete B-box domain at the N-terminus. ThCOL1, ThCOL4, ThCOL6, and ThCOL7 belonged to the third group that contained a complete B-box domain and a B-box domain with secondary structure changes at the N-terminus (Figure 2).

### Expression Patterns of *ThCOL* Genes in Plants Under Several Abiotic Stresses and Treated With ABA

Quantitative real-time PCR was used to analyze the expression patterns of *ThCOL* genes in plants subjected to NaCl, PEG_6_,_000_, and CdCl_2_ stress and ABA treatments to investigate the functions of the *ThCOL* genes.

**FIGURE 3:**
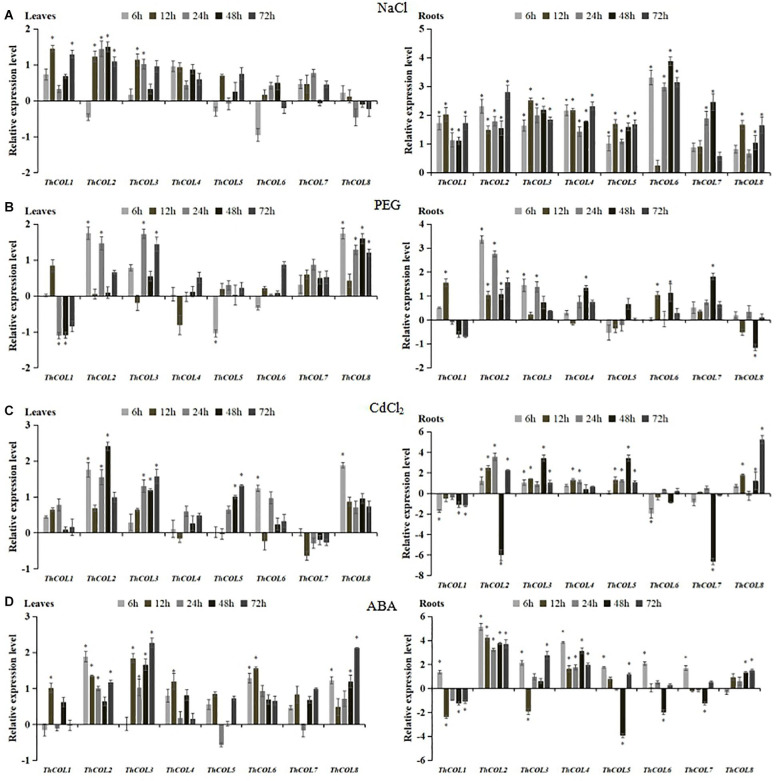
Expression analysis of *ThCOLs* responding to NaCl, PEG_6_,_000_, CdCl_2_, and ABA stresses in *T. hispida* roots and leaves. All relative transcription levels were log_2_-transformed. The error bars were obtained from multiple replicates of quantitative real-time PCR (qRT-PCR). *Represents a significant difference (*P* < 0.05).

In plants under NaCl stress, the expression of *ThCOL4*, *5*, *6*, *7*, and *8* did not change significantly in leaves. *ThCOL1* and *ThCOL3* were significantly upregulated at only two time points (12 and 72; 12–24 h, respectively) of stress exposure. The expression of *ThCOL2* was significantly upregulated at 12–72 h of stress exposure. In roots, the expression of *ThCOL1*, *2*, *3*, *4*, and *5* was significantly upregulated at all time points of stress exposure, in which the expression of *ThCOL2* peaked at 72 h, with a value that was 6.99 times higher than the control. The expression levels of *ThCOL6*, *7*, and *8* at one (12 h), three (6, 12, and 72 h), and two time points (6 and 24 h) of stress exposure, respectively, were not significantly changed, and the expression of these genes was significantly increased at other time points of stress exposure (Figure 3A).

The expression of *ThCOL4*, *5*, *6*, and *7* did not change significantly in leaves under PEG_6_,_000_ stress (except that *ThCOL5* was downregulated in the early stage of stress). The expression levels of *ThCOL2* and *ThCOL3* were significantly upregulated at two time points (6 and 24; 24 and 72 h, respectively) of stress exposure, but no significant changes were observed at the other time points. In contrast, the expression of *ThCOL1* was significantly downregulated at two time points (24 and 48 h) of stress exposure, and no significant changes were observed at other time points. *ThCOL8* expression was significantly upregulated at most of all time points of stress exposure (except 12 h). In roots, the expression of *ThCOL1*, *3*, *4*, *6*, *7*, and *8* did not change significantly at three or four time points of stress exposure. No significant change in *ThCOL5* expression was observed at any time point of stress exposure. *ThCOL2* expression was significantly upregulated at all time points of stress exposure, and *ThCOL2* was able to produce a rapid response to PEG_6_,_000_ stress that was 10.22 times higher than the control at 6 h (Figure 3B).

In plants under CdCl_2_ stress, the expression of *ThCOL1*, *4*, and *7* in leaves was not significantly altered. The expression of *ThCOL2* and *ThCOL3* was upregulated at three time points (6, 24, and 48; 24–72 h, respectively) of stress exposure. *ThCOL5* expression did not change significantly in the early stage of stress but increased significantly at 48–72 h. The expression levels of *ThCOL6* and *ThCOL8* were upregulated at 6 h, but were not significantly change at 12–72 h. In roots, the expression of *ThCOL4* was significantly upregulated at 12–24 h of stress exposure, the expression of *ThCOL6* and *7* was significantly downregulated at one time point (6 and 48 h, respectively) of stress exposure. *ThCOL1* expression was significantly downregulated at 6, and 48–72 h of stress exposure, while *ThCOL3*, *5*, and *8* expression levels were significantly upregulated or remained unchanged at all time points. Notably, *ThCOL2* expression increased gradually from 6 to 24 h, decreased sharply at 48 h to only 1.5% of the control level, and increased significantly at 72 h (Figure 3C).

After the ABA treatment, the expression of *ThCOL2*, *3*, and *8* in leaves was significantly increased or remained unchanged at all time points. The expression of *ThCOL1*, *4*, and *6* were upregulated at 12 h of stress exposure. The expression of *ThCOL5* and *7* did not change significantly at all time points of stress exposure. In roots, the expression of all *ThCOL* genes was divided into two groups with distinct expression trends. The first group included *ThCOL1*, *5*, *6*, and *7*, the expression of which was significantly inhibited at 48 h of stress exposure, the *ThCOL3* expression was significantly inhibited at 12 h of stress exposure. In particular, *ThCOL5* expression was the most obviously inhibited gene at 48 h, and the expression level was only 6.6% of the control. The expression of the second group (*ThCOL2*, *4*, and *8*) was significantly increased or remained unchanged at all stress time points, among which *ThCOL2* and *ThCOL4* potentially respond early to ABA treatment and reached peak expression at 6 h; the expression was 35.03 and 14.44 times higher than the control, respectively (Figure 3D).

Based on these results, all 8 *ThCOL* genes responded to three abiotic stresses (salt, drought and heavy metals) and ABA treatment. Notably, in plants exposed to PEG_6_,_000_, CdCl_2_, and ABA treatments, the expression of *ThCOL2* in roots changed most significantly. The expression of *ThCOL2* in the roots and leaves of *T. hispida* under NaCl stress was significantly upregulated at various time points. Thus, *ThCOL2* may play a more important role in the process by which plants respond to abiotic stress. Therefore, the salt tolerance function and characteristics of *ThCOL2* were further studied.

### *ThCOL2* Is Localized in the Nucleus and Exhibits Transactivation Activity

**FIGURE 4:**
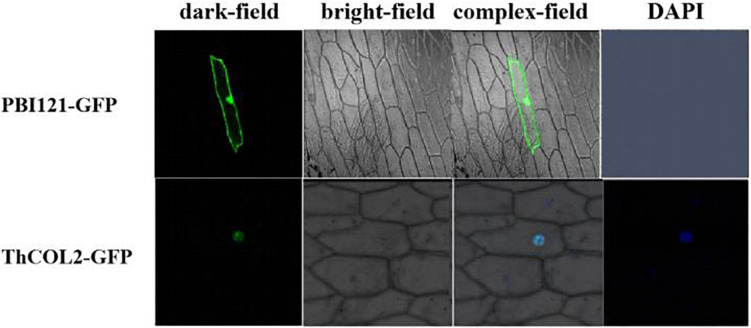
Nuclear localization of ThCOL2 proteins. The *ThCOL2*-GFP fusion gene and GFP (control) were transiently expressed in onion epidermal cells by the particle bombardment (Bio-Rad, Hercules, CA, United States) method. The transformed cells were cultured on MS medium for 2–3 days and visualized using a confocal microscope at 488 nm for excitation of GFP and 507 nm longpass for emission (LSM410, Zeiss, Jena, Germany).

**FIGURE 5:**
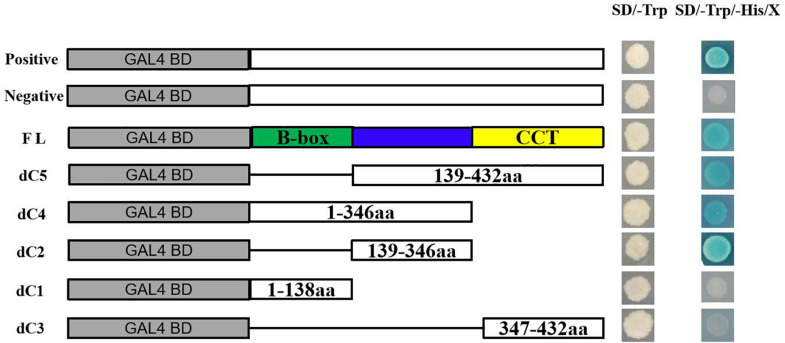
Transcriptional activation assays of the full-length ThCOL2 protein and truncated versions lacking different protein domains. This approach uses the five-segment method to make a deletion, dividing the full-length protein into three segments (dC1, dC2, and dC3) based on the conserved domain, while the dC4 = (dC1 + dC2) and the dC5 = (dC2 + dC3). This study investigates which these five fragment have transcriptional activation activity and which region was necessary for transcriptional activation. SD/-Trp and SD/-Trp/-His/X indicate SD media lacking Trp and SD media with X-α-Gal added but lacking Trp and His, respectively. Positive control = pGBKT7-53 + pGADT7-T, Negative control = pGBKT7-Lam + pGADT7-T.

Two days after transformation, confocal laser scanning microscopy was used to examine the cells. The confocal image showed that the *ThCOL2*–GFP fusion protein was targeted to the nucleus (Figure 4). The results of the transactivation activity experiment showed that all fusion plasmids allowed Y2H Gold cells to grow normally on SD/-Trp medium after transformation, and pGBKT7–*ThCOL2* (1–432 aa), pGBKT7–dC2 (139–346 aa), pGBKT7–dC4 (1–346 aa), and pGBKT7–dC5 (139–432 aa) allowed cells to grow and show a blue color on the screening medium (SD/-Trp/-His/X-a-Gal) (Figure 5). Based on these results, ThCOL2 has transcriptional activation activity, and the transcriptional activation domain might be located at 139–346 aa. Because dC2 did not contain the complete conserved domain of the ThCOL2 protein, we speculate that the B-box and CCT domains might not play a decisive role in the transcriptional activation activity of the ThCOL2 protein.

### Generation of Transient Expression of *ThCOL2* in *T. hispida*

**FIGURE 6:**
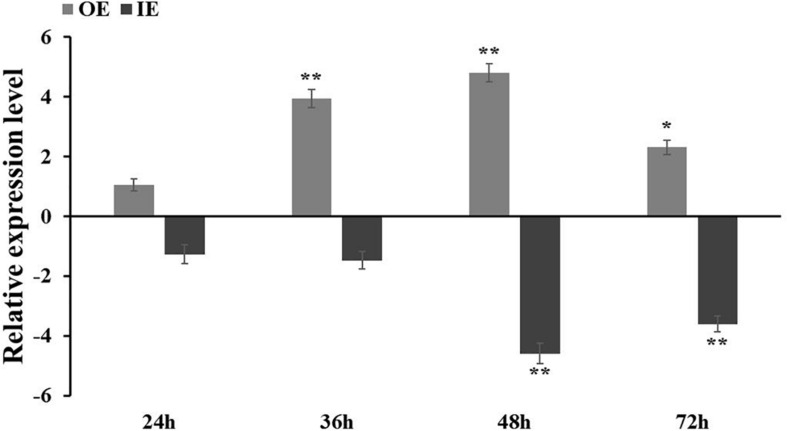
*ThCOL2* transcript levels in *T. hispida* plants with transient overexpression and inhibition expression. The expression data were log_2_ transformed. 1-month-old *T. hispida* plants were transiently transformed with empty pROKII (as control), 35S:COL, pFGC:COL and were then grown on 1/2 MS medium for 24, 36, 48, and 72 h, respectively. The expression of *ThCOL2* in whole OE, IE, and Con plants was measured. Error bars represent standard deviation calculated from multiple qRT-PCR replicates. OE, *ThCOL2* overexpression; IE, *ThCOL2* inhibition expression; Con, pROKII vector control. *Represents a significant difference (*P* < 0.05). **Represents a very significant difference (*P* < 0.01).

The recombinant vectors pROKII–*ThCOL2* and pFGC5941–*ThCOL2* were successfully constructed to further verify whether the *ThCOL2* gene improved salt tolerance. The empty pROKII vector served as a control. The expression level of the *ThCOL2* gene in three transgenic *T. hispida* species after different coculture times was analyzed using qRT-PCR to determine the success of the overexpression and silencing of the *ThCOL2* gene in *T. hispida* and to determine the appropriate time point for transient transformation. Compared with the control, *ThCOL2* expression was significantly increased in OE plants and significantly reduced in IE plants. In particular, at 48 and 72 h of cocultivation, the expression levels of *ThCOL2* in OE plants were 29.8 and 4.95 times higher than the control, respectively. The expression levels of *ThCOL2* in IE plants were 4.1 and 8.2% of the control, respectively (Figure 6). Thus, these plants transiently expressing *ThCOL2* constructs are suitable for further research.

### Physiological Characterization of *T. hispida* With Transient Overexpression and Inhibition of *ThCOL2* Expression Under Salt Stress

**FIGURE 7:**
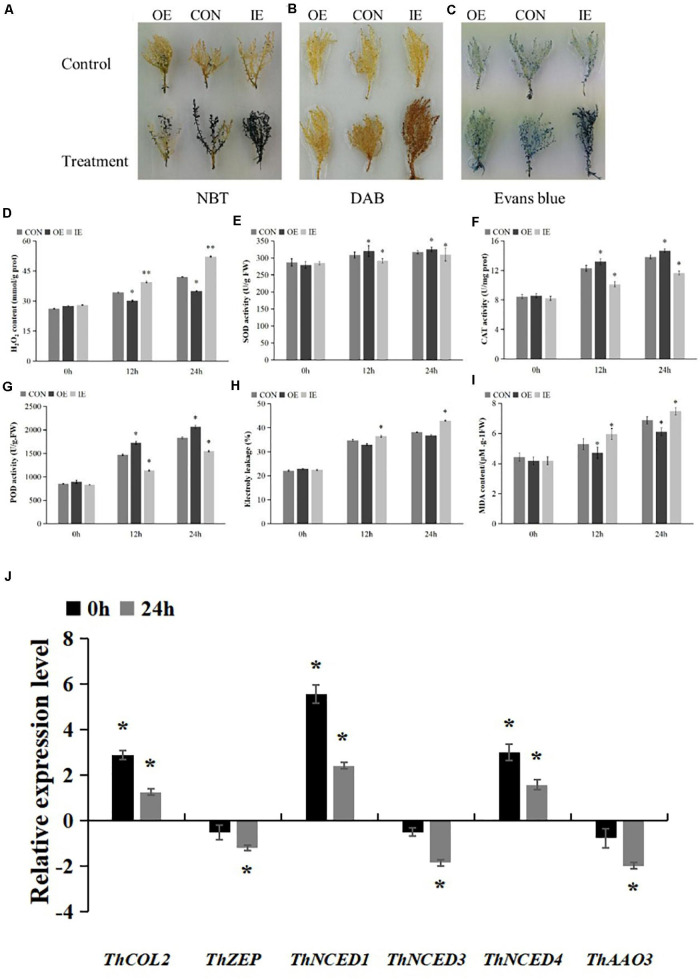
Histochemical staining and the comparison of physiological indicators between transgenic *T. hispida* and control under 150 mM NaCl stress. **(A,B)** The plants were stained with NBT and DAB to reveal the accumulation of O^2–^ and H_2_O_2_, respectively. **(C)** Evans blue staining analysis of cell death. **(D)** Determination of H_2_O_2_ contents. **(E–G)** SOD, CAT, and POD contents analysis the ability of cells to scavenge ROS. **(H)** Analysis of cell death by measurement of electrolyte leakage. **(I)** MDA contents analysis of OE, IE, and Con plants. *Represents a significant difference (*P* < 0.05). **Represents a very significant difference (*P* < 0.01). **(J)** Analysis of the expressions of 5 ABA biosynthesis genes in *T. hispida* plants with transient over-expressioned *ThCOL2*. “0 h” represent after 48 h of coculture, 0 h of salt stress “24 h” represent after 48 h of coculture, 24 h of salt stress. The expression data were log_2_ transformed. *Represents a significant difference (*P* < 0.05). **Represents a very significant difference (*P* < 0.01).

The results of NBT and DAB staining showed that the coloring of the three types of transiently transformed *T. hispida* was basically the same under normal conditions. However, after 2 h of salt stress, the coloration of all three strains obvious increased. Among them, the IE plant was the darkest and the OE plant was the lightest, indicating that the overexpression of *ThCOL2* led to a decrease in the accumulation of O^2–^ and H_2_O_2_ in the cell. The inhibition of *ThCOL2* expression caused the accumulation of intracellular O^2–^ and H_2_O_2_ (Figures 7A,B). Similar results were obtained from the measurement of the H_2_O_2_ content. Under normal conditions, a significant difference in the H_2_O_2_ content was not observed among the three types of transgenic plants (CON, OE, and IE). After exposure to salt stress, the H_2_O_2_ content in OE plants was significantly less than in CON plants. The H_2_O_2_ content of IE plants was significantly higher than CON plants. Specifically, after exposure to NaCl stress for 12 h, the H_2_O_2_ contents of OE and IE plants were 87% and 1.15-fold higher than CON plants, respectively. After 24 h, the H_2_O_2_ contents of OE and IE plants were 83% and 1.24-fold higher than CON, respectively (Figure 7D). This result suggested that the overexpression of the *ThCOL2* gene significantly reduced H_2_O_2_ accumulation in transgenic plant cells under salt stress.

Under non-stress conditions, no significant difference in SOD activity was observed among the three types of transgenic plants. Upon exposure to salt stress, SOD activities increased in OE plants and decreased significantly in IE plants. After 12 h of salt stress, SOD activities in IE and OE plants were 94.6% and 1.04-fold of the values of control plants, respectively. After 24 h of salt stress, SOD activities in IE plants and OE plants were 97.6% and 1.02-fold the values of control plants, respectively (Figure 7E).

Under non-stress conditions, no significant difference in CAT activity was observed among the three types of transgenic plants. Upon exposure to salt stress, CAT activities increased in OE plants and decreased significantly in IE plants. After 12 h of salt stress, CAT activities in IE and OE plants were 82.3% and 1.07-fold, respectively, the values of control plants. After 24 h of salt stress, CAT activities in IE plants and OE plants were 84.3% and 1.06-fold the values of control plants, respectively (Figure 7F).

Under non-stress conditions, no significant difference in POD activity was observed among the three types of transgenic plants. Upon exposure to salt stress, POD activities increased in OE plants and decreased significantly in IE plants. After 12 h of salt stress, POD activities in IE and OE plants were 77% and 1.17-fold, respectively, the values of control plants. After 24 h of salt stress, POD activities in IE plants and OE plants were 75% and 1.12-fold the values of control plants, respectively (Figure 7G). Based on these results, *ThCOL2* enhances ROS scavenging by increasing the activity of SOD, CAT, and POD enzymes.

Normal living cells have a complete membrane structure, preventing Evans blue from entering the cell. However, inactive cells are stained with Evans blue because of the increase in membrane permeability. Moreover, deeper Evans blue staining is observed when more cells with no activity or an incomplete membrane are present. Therefore, Evans blue staining was used to analyze the damage to the plant cell membrane (Liu Z. Y. et al., 2020). Electrolyte leakage was used to detect the extent of cell death. In this study, Evans blue staining showed no obvious difference in the color of the three types of transgenic plants under normal conditions, but after 2 h of NaCl stress, the colors of OE and IE plants were obvious lighter and deeper than the control, respectively (Figure 7C). The electrolyte leakage data further confirmed these results. Under normal conditions, no obvious difference was detected between the three types of transgenic plants. After 12 h of salt stress, the relative conductivities of OE and IE plants were 94% and 1.05-fold the values of Con plants, respectively. After 24 h, the relative conductivities of OE and IE plants were 96% and 1.12-fold the values of Con plants, respectively (Figure 7H).

The analysis of MDA levels showed no difference between the three types of transgenic plants under normal conditions. However, upon exposure to salt stress, the MDA content of OE plants was significantly lower and the MDA content of IE plants was significantly higher than Con plants. After 12 h and 24 h of salt stress, the MDA content of OE plants was 89 and 88% the values of the Con plants, and the MDA content of IE plants was 1.12- and 1.08-fold the values of Con plants, respectively (Figure 7I). Thus, membrane lipid peroxidation of plants overexpressing *ThCOL2* is substantially reduced.

Furthermore, the relative expression levels of five genes related to ABA biosynthesis in the OE plants were measured using qRT-PCR. After 48 h of coculture, the expression levels of *ThNCED1* and *ThNCED4* were significantly higher, and the expression levels of *ThNCED3*, *ThZEP*, and *ThAAO*3 were not significantly altered in OE compared with CON. But after 24 h of salt stress, the expression levels of all five studied genes all were lower than the normal condition (Figure 7J).

## Discussion

Genes in the CO-like transcription factor family have been cloned and characterized in various plants, such as 17 COL proteins in Arabidopsis (Robson et al., 2001), 16 COL proteins in rice (Yano et al., 2000), 9 COL proteins in barley (Griffiths et al., 2003), 3 COL proteins in tomato (Ben-Naim et al., 2006), and 16 COL proteins in corn (Miller et al., 2008). In the present study, we cloned eight single *ThCOL* genes from *T. hispida* with complete ORFs.

CONSTANS-LIKE proteins are divided into three groups according to the B-box domains. A study on Arabidopsis COL protein function revealed that COL transcription factors with similar functions were clustered in similar types. Therefore, according to the phylogenetic grouping, the functions of the *T. hispida ThCOL* genes were predicted. Phylogenetic results showed that the *ThCOL2*, *ThCOL5*, and *ThCOL8* genes were grouped into the first class. AtCOL4, a class I COL transcription factor, regulates tolerance to abiotic stress in an abscisic acid-dependent manner (Min et al., 2015). Steinbach (2019) showed that AtCOL4 also plays a role in suppressing flowering by functioning as a transcriptional repressor of the *FT* gene.

In Arabidopsis, some other COL proteins of the class I family also play roles in flowering and circadian rhythms. For example, overexpression of *AtCOL5* induces flowering in Arabidopsis growing in a short day period (Hassidim et al., 2009). *AtBBX32* interacts with *AtCOL3* to regulate flowering (Tripathi et al., 2017). Ledger et al. (2001) analyzed the circadian rhythms of transgenic plants overexpressing *AtCOL1* and showed that overexpression of *AtCOL1* can shorten two different circadian rhythm cycles. Experiments with the highest expression of *AtCOL1*-overexpressing lines showed that circadian deficiency depends on the fluence rate, suggesting that *AtCOL1* affects the light input pathway. Therefore, we speculated that the *ThCOL2*, *ThCOL5*, and *ThCOL8* genes may participate in the responses to abiotic stress and the photoperiod.

Quantitative real-time PCR was performed to analyze the expression of the 8 *ThCOL* genes in plants exposed to three abiotic stresses and ABA treatments to determine whether the 8 *ThCOL* genes were involved in the stress response in *T. hispida*. All of these genes allowed the plants to respond to one or more of these stress treatments, and their expression patterns varied. And the obvious differential time-course expressions of these eight genes were found. For example, in leaves, upregulation of *ThCOL2* gene under PEG treatment at 6 and 24, but not at 12, 48, and 72 h. At the time, continuously upregulation of *ThCOL2* gene under NaCl treatment at 6–72 h. This phenomenon maybe common in plant, such as *AtCOL4* expression was significantly upregulated after 3 h of NaCl stress, and returned to the control level after 6 h of NaCl stress (Min et al., 2015). While *BnCOL2* expression continued to decrease after 1, 3, and 6 h of NaCl stress in *Brassica napus* (Liu L. D. et al., 2020). We speculate that the expression of some genes may be expressed regularly throughout the time, but some genes have relatively large differences at different time points.

In particular, the expression of the *ThCOL2* gene in the roots and stems of *T. hispida* changed significantly at most time points of exposure to the three abiotic stresses (salt, drought and heavy metals) and ABA treatments. Thus, it may play an important role in the ability of *T. hispida* to resist stress. Especially, under salt stress, *ThCOL2* gene was significantly upregulated at all time points in leaves and roots (except 6 h in leaves).

In this study, both *ThCOL2* and *AtCOL4* are class I COL proteins. Min et al. (2015) showed that mutation of the *atcol4* gene in Arabidopsis resulted in increased sensitivity to ABA and salt stress during seed germination and cotyledon regeneration, while overexpression of the *AtCOL4* gene reduced plant sensitivity to ABA and salt stress. Moreover, in *AtCOL4*-overexpressing and wild-type plants under ABA or salt stress, the transcript levels of other ABA biosynthesis- and stress-related genes were increased. Based on these results, *AtCOL4* participates in ABA and salt stress responses through ABA-dependent signaling pathways.

The qRT-PCR results revealed significantly increased expression of *ThCOL2* in the roots and stems of *T. hispida* under NaCl and ABA stress at most time points. Given this consistent trend, we speculate that *ThCOL2* is also involved in the ABA pathway-related response to NaCl stress. The relative expression levels of the genes related to ABA biosynthesis in the *ThCOL2* OE transgenic lines were measured using qRT-PCR, and the results indicated that the *ThCOL2* gene might regulate the expression of some ABA biosynthesis genes. These findings will be further verified in future studies.

*Tamarix hispida* is a woody halophyte with a very high salt tolerance. Therefore, the salt tolerance function of the *ThCOL2* gene was preliminarily and quickly identified through transient transformation. Histochemical staining and physiological index measurements showed that overexpression of *ThCOL2* enhanced the salt tolerance of transgenic plants by increasing the activity of protective enzymes, reducing the accumulation of ROS and MDA in plants, and reducing cell damage. Interestingly, *OMG1*, a Arabidopsis class III COL protein, has played a role in regulating ROS pathway function by regulating the expression of genes involved in the ROS pathway (*MYB77* and *GRX480*) (Sng et al., 2018). *ThCOL2* overexpression also regulates the accumulation of ROS in plants to reduce damage caused by salt stress, indicating that class I and III COL proteins all play a certain role in regulating ROS pathway function. Whether the temporal expression changes of *ThCOL2* in response to salt stress affects its physiological regulation function, and the relationship between them will further analysis through stable transform *ThCOL2* into *T. hispida.*

The CCT domain of the COL protein is involved in nuclear localization and protein-protein interactions (Kurup et al., 2000). In this study, the ThCOL2 protein was localized in the nucleus. Moreover, the ThCOL2 protein exhibited transcriptional activation activity. Therefore, the ThCOL2 protein has the general characteristics of a transcription factor. An analysis of the transcriptional activation domain of the ThCOL2 protein revealed that the transcriptional activation domain of the protein is located in the middle segment between the B-box and CCT domains, consistent with the results of a previous study by Min et al. (2015).

In summary, 8 *ThCOL* genes were cloned from *T. hispida* in this study, and the expression patterns of these genes in response to various stresses were analyzed. The salt tolerance function of *ThCOL2* was further studied by transient transformation, and the salt tolerance mechanism and protein characteristics were preliminarily analyzed. In subsequent studies, we will further transform *ThCOL2* into *T. hispida* or other model plants and analyze the salt tolerance function and its regulatory mechanism in transgenic plants.

## Conclusion

The CO-like transcription factor is a key transcription factor that plays an important role in regulating plant flowering. In addition, it is also involved in the abiotic stress response of plants. In this study, 8 *ThCOL* genes were identified in *T. hispida*. *ThCOL* gene expression was obviously altered in response to abiotic stress (salt, osmotic and heavy metal stress) and ABA treatment. Further experiments performed using the *ThCOL2* gene, which responded significantly to salt stress. *ThCOL2* is located in the nucleus and has transcriptional activation activity. The transcriptional activation domain is located at 139–346 aa. Moreover, overexpression of *ThCOL2* in *T. hispida* under salt stress not only enhanced the ROS scavenging capacity to reduce cell damage and death caused by salt stress but also increased the activity of protective enzymes. Conversely, transiently transformed plants with a construct that inhibited *ThCOL2* expression displayed the opposite physiological changes. Thus, *ThCOL2* effectively enhances the tolerance of transgenic *T. hispida* to salt stress.

## Data Availability Statement

The datasets presented in this study can be found in online repositories. The names of the repository/repositories and accession number(s) can be found in the article/Supplementary Material.

## Author Contributions

XL and BT wrote the manuscript and performed some of the assays. JL and ZL performed the assays. JW performed the data analysis. CG provided funds for the current study, designed the study, and revised the manuscript. All authors contributed to the article and approved the submitted version.

## Conflict of Interest

The authors declare that the research was conducted in the absence of any commercial or financial relationships that could be construed as a potential conflict of interest.

## Footnotes

1 www.blast.ncbi.nlm.nih.gov/Blast.cgi

2 https://www.ncbi.nlm.nih.gov/orffinder/

3 https://web.expasy.org/protparam/

## Supplementary Material

Supplementary Figure 1 Construction of pFGC5941-*ThCOL2* vector.

## Abbreviations

NBT: nitrotetrazolium blue chloride
DAB: diaminobenzidine
SOD: superoxide dismutase
CAT: catalase
POD: peroxidase
MDA: malondialdehyde
FT: LOCUS T
Ghd7: grain height date 7
Ehd1: early heading date 1
OsRACK1: receptor for activated C-kinase 1
COL: CONSTANS-LIKE
Ghd2: grain height date 2.
