# Supplementary figures and images for: ThCOL2 Improves the Salt Stress Tolerance of Tamarix hispida
Source: Front Plant Sci. 2021 May 17;12:653791. doi: 10.3389/fpls.2021.653791 (PMC8166225; doi:10.3389/fpls.2021.653791)

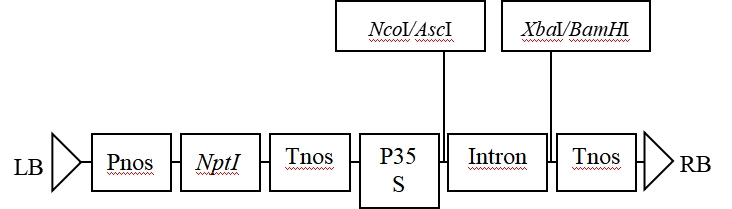

Supplement: Supplementary Figure 1 — Construction of pFGC5941-ThCOL2 vector. [file Image_1.jpg]
