# Supplementary material for: ThCOL2 Improves the Salt Stress Tolerance of Tamarix hispida
Source: Front Plant Sci. 2021 May 17;12:653791. doi: 10.3389/fpls.2021.653791 (PMC8166225; doi:10.3389/fpls.2021.653791)
Supplement: Supplementary file 2 [file Table_1.docx]

**Table S1**

**Primers sequences and amplification conditions**

| **Forward and reverse primers (5′–3′)** | | | |
| --- | --- | --- | --- |
| **Genes** | **Primers used for real-time RT-PCR** | | |
| *ThCOL1* | GACGTACAAACGGTGGAT | | ATACCCTGACGATTGGAAT |
| *ThCOL2* | GGCGATGAATGTTTCAGT | | GCATAGCATACTGCTGTT |
| *ThCOL3* | GACATCAACTGCTTTGTTCC | | ATAGCTTCCATGCTTCCT |
| *ThCOL4* | GGTGAGAATGCTGTTGCT | | GCAGCCCACAAGACAAAT |
| *ThCOL5* | ATGTTGATGTGGATACCTGGCT | | CGTAACACGAGAACTTGCAGCT |
| *ThCOL6* | TGGCAGTGATTAAGCAGCTT | | ACCAACTGTACGCCCATT |
| *ThCOL7* | CGGATGGAGTCCGTACCACAAT | | AGGCTGTGTGGCATTAGATAGT |
| *ThCOL8* | GTGGCGATGTGGATGACT | | GGCTAATTGATGCGTTGT |
| *Actin* | AAACAATGGCTGATGCTG | | ACAATACCGTGCTCAATAGG |
| *α-tubulin* | CACCCACCGTTGTTCCAG | | ACCGTCGTCATCTTCACC |
| *β-tubulin* | GGAAGCCATAGAAAGACC | | CAACAAATGTGGGATGCT |
| **Constructs** | **Primers used in constructing plant plasmids** | | |
| pROKII-*ThCOL2* | CGCGGATCCATGACAATCGAGTCTCCTTCAC | | CGAGCTCTTACTTCTGACACGTGATGACT |
| pROKII | AGACGTTCCAACCACGTCTT | | CCAGTGAATTCCCGATCTAG |
| pFGC5941-*ThCOL2* | *ThCOL2*-Sense | | |
|  | CATGCCATGGCTTCTTGGCCCCGAAAGGCGAT | | TTGGCGCGCCCCTCCATAACTCTTATGTGG |
|  | *ThCOL2*-Anti | | |
|  | GCTCTAGACTTCTTGGCCCCGAAAGGCGAT | | CGCGGATCCCCTCCATAACTCTTATGTGG |
| Sense | ATAAGGAAGTTCATTTCATTTG | | CAATCAAATGAAGAGCCAAT |
| Anti | CTTACTTACACTTGCCTTGGAG | | ATCTGAGCTACACATGCTCAG |
|  | **Primers used in subcellular localization analysis** | | |
| pBI121-*ThCOL2*-GFP | GCTCTAGAATGACAATCGAGTCTCCTTC | | GGACTAGTCTTCTGACACGTGATGACT |
| pBI121-GFP | TTTCATTTGGAGAGAACACG | | CGACCAGGATGGGCACCAC |
|  | **Primers used in yeast experiment analysis** | | |
| pGBKT7-*ThCOL2*-FL | CCGGAATTCATGACAATCGAGTCTCCTTC | | CGCGGATCCTTACTTCTGACACGTGATGACT |
| pGBKT7-*ThCOL2*-dC1 | CCGGAATTCATGACAATCGAGTCTCCTTC | | CGCGGATCCGATGGGAACCCGGTGGT |
| pGBKT7-*ThCOL2*-dC2 | CCGGAATTCCACCCCATCGTCGGCGGCAT | | CGCGGATCCATCCACTGGAGCGAGCT |
| pGBKT7-*ThCOL2*-dC3 | CCGGAATTCAGGGAGGCTAGAGTTCT | | CGCGGATCCTTACTTCTGACACGTGATGACT |
| pGBKT7-*ThCOL2*-dC4 | CCGGAATTCATGACAATCGAGTCTCCTTC | | CGCGGATCCATCCACTGGAGCGAGCT |
| pGBKT7-*ThCOL2*-dC5 | CCGGAATTCCACCCCATCGTCGGCGGCAT | | CGCGGATCCTTACTTCTGACACGTGATGACT |
| pGBKT7 | TCATCGGAAGAGAGTAGT | | AGAGTCACTTTAAAATTTGTAT |
|  | **Primers used in ABA biosynthesis analysis** | | |
| ThNCED3 | ATTTCTTCGACGGCGATGGC | GTGATCAAGCAGGCCAAACAG | |
| ThAAO3 | ATGTATCTTGATCGCAAGACTG | AAGATAATGCACCCCAATC | |
| ThZEP | AGGTGATCTTCTTGTTGGAG | CAACGCCACCAGCAGGTTCCTT | |
| ThNCED1 | AGATGAAATGGGTGGAGGT | GATCATCTTGGCTGAGAAT | |
| pdbNCED4 | CTGCATCTATATCCGCAGT | GTTAGCTAGTCCGATGCCAT | |
